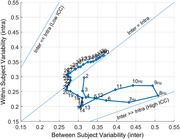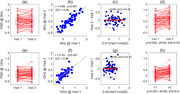# Inter‐ and intra‐subject variability of quantitative EEG biosignatures and their effect on interpretation of normalized effect size

**DOI:** 10.1002/alz.084211

**Published:** 2025-01-09

**Authors:** Amir H. Meghdadi, Chris Berka, Michael H. Malek‐Ahmadi

**Affiliations:** ^1^ Advanced Brain Monitoring, Carlsbad, CA USA; ^2^ Advanced Brain Monitoring, Inc., Carlsbad, CA USA; ^3^ Banner Alzheimer's Institute, Phoenix, AZ USA; ^4^ University of Arizona College of Medicine‐Phoenix, Phoenix, AZ USA

## Abstract

**Background:**

Quantitative EEG measures can be used as biosignatures of disease conditions. As such, the effect of interventions/treatments can be studied by longitudinal analysis of changes in these measures. The consistency of these measures can be assessed by test‐retest reliability scores such as intra‐class correlation coefficient (ICC) that depends on intra‐ and inter‐subject variability. The magnitude of an effect can be described by a normalized effect size (i.e. normalizing the effect with respect to the sample variability). However, the inherent variability of EEG and its effect on interpretation of effect size is less explored. The aim of this work is to investigate inter‐ and intra‐subject variability of PSD‐based resting‐state EEG measures and their effect on analysis of change scores.

**Methods:**

We collected 20‐channel longitudinal EEG data (initial visit and 1‐year follow‐up) from healthy volunteers (n=61, ages 40‐82). After artifact decontamination, we computed power spectral density (PSD) at 1‐40 Hz frequency bins. At each frequency, we computed inter‐ and intra‐subject variability of PSD as well as ICC. We simulated a hypothetical effect by adding a constant value to the 2nd visit’s data equivalent to half the standard deviation of the sample. We computed the normalized effect size (Cohen’s d) using both the pooled variance as well the variability in the change score. We compared these effect sizes for different frequencies.

**Results:**

Overall, absolute PSD exhibited high test‐retest reliability in the frequencies 2‐20 Hz (ICC>0.7) with 7‐9 Hz having the highest ICC (ICC>0.94). Inter‐subject variability was highest at 8Hz, and intra‐subject variability was lowest at 13‐15 Hz frequencies. For frequencies between 5 – 10 Hz (fast‐Theta/slow‐Alpha bands), the difference between the two types of normalized effect size ranged between 0.3 to 0.6.

**Conclusions:**

Inter‐ and intra‐subject variability of PSD‐based EEG measures depend on frequency. As such, changes in PSD due to potential interventions should be interpreted with respect to the inherent variability of PSDs. In longitudinal analysis of paired data, normalized effect size should be reported using both population variability (inter‐subject) and change‐score variability (intra‐subject).